# Investigation of the antimycobacterial potential and toxicity evaluation of a proteinaceous compound from *Streptomyces qinglanensis* VITABS23 against *Mycobacterium tuberculosis* strains

**DOI:** 10.1186/s41182-025-00859-6

**Published:** 2025-12-19

**Authors:** Apsara S. Babu, K. V. Bhaskara Rao

**Affiliations:** Marine Biotechnology Laboratory, Department of Biomedical Sciences, School of Biosciences and Technology, Vellore Institute of Technology, Vellore, Tamil Nadu India

**Keywords:** Marine actinobacteria, Antimycobacterial activity, Protein purification, Tuberculosis, Acute toxicity, Sub-acute toxicity

## Abstract

**Background:**

Tuberculosis (TB), an infectious disease caused by *Mycobacterium tuberculosis,* remains a major public health challenge. The emergence of drug-resistant strains has further limited effective treatment options. Therefore, discovering novel antimycobacterial agents from underexplored habitats is essential. In this study, the marine actinobacterial extract *Streptomyces qinglanensis* VITABS23, isolated from mangrove sediments was evaluated for its antimycobacterial activity. The active protein was extracted and identified as a potential therapeutic candidate, and its toxicity profile was evaluated in animal models.

**Methods:**

The cell-free extract of *Streptomyces qinglanensis* VITABS23 was screened for antimycobacterial activity against *M. tuberculosis* strains using agar well diffusion and microplate Alamar blue assays. Aqueous extracts were precipitated with 70% ammonium sulphate, dialyzed and purified by DEAE Sepharose ion exchange chromatography. The purified protein was characterized by sodium dodecyl sulphate polyacrylamide gel electrophoresis (SDS PAGE) and MALDI TOF analysis. For toxicity evaluation, in vivo studies were carried out in albino Wistar rats to determine the safety profile through acute (single doses of 100 and 350 mg/kg body weight) and sub-acute (repeated oral dosing) studies following OECD guideline 423. Body weights, hematological and biochemical parameters, and organ histopathology were assessed at the end of the experimental period.

**Results:**

The extract showed maximum inhibition zones against *M. smegmatis* (26 mm) and *M. tuberculosis* H37Ra (22 mm) at 50 mg/ml. Minimum Inhibitory Concentration assays showed strong activity at a 500 µg/mL concentration, with 85% inhibition for *M. tuberculosis* H37Ra and 78% for *M. smegmatis*. Ion exchange chromatography yielded a 156-fold purification with a protein yield of 0.085% and a specific activity of 4166 IU/mg. The active protein had an intact molecular weight of 20 kDa. Acute toxicity studies showed no adverse effects at doses of 100 and 350 mg/kg body weight. Sub-acute studies with repeated dosing for 28 days revealed no mortality, toxic symptoms or significant differences compared with controls. Histopathological analysis of the vital organs in both studies revealed normal tissue architecture suggesting no morphological changes.

**Conclusion:**

The *S. qinglanensis* VITABS23 extract from mangrove sediments demonstrates potent antimycobacterial activity and a favorable safety profile, highlighting its potential as a candidate for tuberculosis treatment.

**Supplementary Information:**

The online version contains supplementary material available at 10.1186/s41182-025-00859-6.

## Background

Tuberculosis (TB), a highly contagious bacterial infection caused by *Mycobacterium tuberculosis* still continues to persist as a global public health concern. In terms of mortality, it is one of the world’s second leading infectious diseases, following the COVID-19 pandemic [1]. According to the World Health Organization (WHO) report (2024), approximately 10.8 million people are infected with TB worldwide with 1.6 million deaths, of which 25–30% of the deaths are related to HIV-associated TB [2]. These figures represent a rapid surge from the previous records of 1.5 million cases and 1.4 million deaths reported in 2019 to 2020. As per the Global Tuberculosis Report 2024, India marks the highest burden of TB cases in 2023, contributing 26% of the global estimate [2, 3].

Before the development of antimycobacterial drugs, tuberculosis was presumed to be an untreatable and lethal disease [4, 5]. The introduction of front-line antibiotics such as rifampicin, isoniazid, ethambutol, streptomycin and pyrazinamide has brought the TB burden under control to some extent [6, 7]. However, the major drawback of these drugs is that they are not patient compliant due to their long-term therapy of more than six months which often poses side effects. Later, the establishment of Bacille Calmette–Guerin (BCG) in combination with these antibiotics has reduced mortality to an extent and treatment duration from 18 to 6 months [4]. But, the emergence of multi-drug resistant TB (MDR-TB) and extensively drug-resistant TB (XDR-TB) has heightened the seriousness of the current situation worrisome. Several second-line injectables (amikacin, bedaquiline, linezolid, kanamycin, aminoglycosides, and capreomycin) were then introduced to treat the intolerant TB patients [6, 8–10]. Even though, these drugs had a profound impact on treating active tuberculosis, their prolonged treatment duration, high medical cost, lower efficacy and higher toxicity profiles pose a significant challenge to the scientific community in curbing this chronic infection [11]. In fact, identifying new compounds effective against *M. tuberculosis* is technically challenging. A prime reason for this is the low permeability of its complex cell wall to antibiotics and hydrophilic molecules making their transport into the cell ineffective [4, 12]. Secondly, multiple efflux pumps embedded in the cell membrane flush out various antimycobacterial drugs thereby decreasing their concentration and effectiveness within the cell [13, 14]. These factors make the development of antimycobacterial drugs difficult. Hence, it is indispensable to identify novel compounds with fewer side effects and better pharmacokinetic properties, capable of targeting various strains of *M. tuberculosis.*

The quest for new antimycobacterial compounds from unexplored sources has become a prime challenge for drug discovery and development. The marine biodiversity is encompassed with diverse microorganisms capable of producing novel bioactive compounds of clinical importance [15]. Among marine habitats, mangroves are specialized aquatic environments occupied in the transition zone of backwaters, estuaries and lagoons of the tropical and subtropical domains [16, 17]. The peculiar environmental conditions like high salinity, low temperature, elevated pressure, high pH and high amounts of organic matter foster the microbial community thriving here to produce unique secondary metabolites with distinct biological activities [18]. Marine actinobacteria found in mangrove sediments are largely untapped and are hidden treasures of new antimycobacterial agents. According to reports, these Gram-positive filamentous bacteria are prolific producers of more than 10,000 bioactive compounds, the majority of them from the genus *Streptomyces* [3, 19, 20]. In fact, actinobacteria-derived bioactive compounds have played an important role in tuberculosis treatment, most notably the discovery of Streptomycin from *Streptomyces griseus*, which marked a major breakthrough in TB therapy [15, 21]. Likewise, several antimycobacterial drugs like rifampicin, kanamycin, viomycin and capreomycin were also obtained from actinobacteria, marking their role in drug discovery. Until now, these compounds continue to be the key players in the treatment of TB [3, 15, 22].

The majority of secondary metabolites derived from actinobacteria are small molecules, while studies on proteinaceous antimycobacterial agents from marine actinobacteria remain limited. Unlike, small-molecule antibiotics (e.g., rifampicin, kanamycin), proteinaceous compounds belong to the class of antimicrobial peptides (AMPs) or non-ribosomal peptides (NRP). Compared with conventional small-molecule antibiotics, proteinaceous antimycobacterial agents exhibit novel mechanisms of action, such as disrupting cell membranes, hydrolyzing cell wall components, modulating immune responses and interacting with intracellular targets like DNA, RNA and proteins [23]. They also demonstrate reduced resistance potential, higher specificity and lower host toxicity in vivo [24]. Examples of proteinaceous antimycobacterial agents from marine actinobacteria include actinomycins, ilamycin G, atratumycin, mollemycin A, and capreomycin [23, 25]. Despite these promising attributes, proteinaceous antimycobacterial agents from mangrove-derived actinobacteria remain underexplored, representing a critical gap in drug discovery. Hence, marine actinobacteria are considered ideal sources of novel drug leads to tackle this infectious disease. Several studies have focused on the isolation of bioactive compounds from mangrove actinobacteria with broad antimicrobial activities [26–29]. However, their potential specifically against *Mycobacterium tuberculosis* has been far less investigated. The present study aims to screen the marine actinobacterial extract, *Streptomyces qinglanensis* VITABS23 from mangrove sediments of Kerala, and to evaluate its antimycobacterial potential against *M. tuberculosis* strains, followed by the extraction and identification of the active protein as a potential candidate for tuberculosis treatment. Additionally, the toxicity profile of *S. qinglanensis* extract was also assessed using animal models.

## Materials and methods

### Chemicals used

All the media and analytical grade chemicals used in this study were purchased from HiMedia Laboratories, Pvt. Ltd., Mumbai, India and Sisco Research Chemicals Pvt. Ltd, India. Diethylaminoethyl (DEAE) Sepharose Fast Flow resin was procured from Sigma Aldrich, USA, and a broad range protein ladder was obtained from ThermoFisher Scientific, Waltham, USA.

### Marine actinobacteria strain

*Streptomyces qinglanensis* VITABS23 was isolated from the mangrove sediments of Munroe Island (9° 0′ 0′′ N, 76° 37′ 0′′ E), Kollam, Kerala, India. The 16S rRNA gene sequence of the strain has been deposited in the GenBank database under the accession number OR7894889.1.

### In vitro antimycobacterial activity of marine actinobacteria extract

#### Bioactive metabolite production

The isolates were inoculated into soluble starch (SS) production broth supplemented with 1 mL of trace elements (0.5% FeSO_4_, CuSO_4_, ZnSO_4_ and MnCl_2_). The pH of the medium was adjusted to 7.5 prior to inoculation. After inoculation, the flasks were incubated in a shaking incubator at 28 °C for 5–6 days. The cultures were then centrifuged at 10,000 rpm for 15 min at 4^o^ C and the cell-free extracts were collected.

### Primary screening

The cell-free extract was evaluated for its antimycobacterial activity using the agar well diffusion method. The bacterial strains *Mycobacterium smegmatis* mc (2)155 (ATCC 700084) and *M. tuberculosis* H37Ra (ATCC 27294) were used as test organisms. The strains were obtained from the Centre for Drug Discovery and Development (CDDD), Sathyabama Institute of Science & Technology, Chennai, India. A loopful of the culture was inoculated into sterilized Middlebrook 7H9 broth base and incubated at 37 °C for 48 h. Following incubation, the turbidity of the culture was adjusted according to McFarland standards and absorbance was measured at 600 nm using a spectrophotometer [30]. The bacterial inoculums were then seeded on Middlebrook 7H9 agar plates using sterile cotton swabs. Wells of 10 mm diameter were made on the agar surface using a sterile cork borer and 100 µL of the cell-free extract at various concentrations (10, 25 and 50 mg/mL) was added into each well. The plates were then incubated at 37 °C for 24 h and the diameter of zones of inhibition was measured. Rifampicin was used as a positive control and dimethyl sulfoxide (DMSO) served as a negative control [7].

### Secondary screening

#### Microplate Alamar Blue Assay (MABA)

The minimum inhibitory concentration of the extract was determined using the Microplate Alamar Blue Assay (MABA) in a sterile 96-well plate. To each well, 100 µL of the extract dissolved in Middlebrook 7H9 broth at concentrations of 500, 250 and 125 µg/mL was added. Subsequently, 100 µL of *M. smegmatis* or *M. tuberculosis* H37RA suspension was added into all the wells, except the negative control wells. The negative control wells contained 200 µL of the broth alone, while the growth control wells contained 100 µL of broth and the bacterial suspension. For the DMSO control, 100 µL of 5% DMSO in Middlebrook broth was added and the drug control wells contained 100 µL of rifampicin (2 µg/mL). The plate was then incubated at 37 °C for 24 h. After incubation, 32.5 µL of Alamar blue dye was added to the growth control wells and the plate was further incubated for 24 h. Once a color change from blue to pink was observed in these wells, 32.5 µL of the dye was added to all remaining wells, followed by an additional 24-h incubation. The plates were then observed for any color changes and the percentage growth inhibition of *Mycobacteria* strains was calculated [31, 32]. The assay was carried out in triplicate.

### Extraction and purification of the antimycobacterial compound

#### Solvent–solvent extraction

The cell-free extract of *Streptomyces qinglanensis* VITABS23, identified as a potential antimycobacterial candidate, was subjected to a conventional solvent–solvent extraction method. The solvents (ethyl acetate, chloroform and hexane) were selected based on their polarity. For extraction, the cell-free extract was mixed with an equal volume of each solvent in a 1:1 ratio. The mixtures were well agitated for a few minutes and then transferred to a separating funnel to allow phase separation into organic and aqueous layers [33, 34]. The aqueous layer containing the metabolites was collected and lyophilized (Alpha 1–2 LSCbasic—Martin Christ) for further purification studies. The dried extracts were subsequently tested for their antimycobacterial activity as described earlier.

#### Ammonium sulphate precipitation and dialysis

The supernatant was precipitated by sequentially adding 0–70% solid ammonium sulphate according to the ammonium sulphate saturation table. The reaction was carried out in ice with constant stirring until complete saturation. After reaching the desired saturation, the solution was maintained at 4 °C for 8–12 h. Further the solution was centrifuged at 8000 rpm for 15 min at 4 °C. Following centrifugation, the precipitated protein pellets were suspended in 50 mM Tris–HCl buffer solution (pH 8) [35, 36] and analyzed for antimycobacterial activity. The sample was further dialyzed using a pretreated dialysis bag (Dialysis membrane-150, HiMedia) suspended in 50 mM Tris–HCl buffer (pH 8) to remove the excess salt present in the sample.

#### Ion exchange chromatography (IEX)

The dialyzed protein sample was purified by ion exchange chromatography using a diethylaminoethyl (DEAE) Sepharose Fast Flow resin. The activated resin was packed into a column of 15 cm in length and 5 cm in diameter. The column was equilibrated with 50 mM Tris–HCl binding buffer (pH 8) [33]. The protein sample was then loaded onto the column, and unbound proteins were washed out with 50 mM Tris–HCl wash buffer (same buffer used for equilibration). Subsequently, the bound proteins were eluted stepwise with elution buffers containing increasing concentrations of NaCl (100 mM, 200 mM, 300 mM, 500 mM, 700 mM and 1 M) prepared with 50 mM Tris–HCl buffer. Fractions of 2 mL each were collected at a flow rate of 500 µL/min [35, 37]. The eluted fractions were quantified for their bioactivity and subjected to protein estimation using modified Lowry’s method [38].

### Estimation of protein content and yield

The protein fractions were subjected to protein estimation by Lowry’s method with slight modifications [38–40]. Bovine serum albumin (BSA) standard solutions were prepared and diluted to various concentrations. A volume of 100 µL of protein solution was mixed with 25 µL of freshly prepared copper tartrate solution in a 96-well microtiter plate [35]. The solutions were properly mixed and incubated for 10 min at room temperature. Following incubation, 10 µL of Folin–Ciocalteu reagent was added, mixed thoroughly and allowed to stand for 30 min at room temperature for color development. Blank was set and the absorbance was read at 630 nm using a spectrophotometer.

### Characterization of the antimycobacterial compound

#### Determination of molecular mass using SDS-PAGE

SDS-PAGE analysis was performed to determine the molecular mass of the purified protein according to the procedure of Laemmli with slight modifications [41]. The active antimycobacterial fraction (200 mM) from ion exchange chromatography was run on a 12% acrylamide gel in Tris glycine buffer of pH 8.3. The proteins were diluted in SDS loading buffer containing β- mercaptoethanol and denatured by heating at 95 °C for 5 min. The proteins and broad range protein ladder were then loaded on the acrylamide gel and electrophoresed at 100 V for 1.5 h. Following electrophoresis, the gel was stained with 0.25% Coomassie brilliant blue R-250 for 3 h [33]. After staining, the gel was repeatedly destained and the protein bands were visualized using the Bio-Rad gel imaging system (Bio-Rad Laboratories, USA).

#### MALDI-TOF MS analysis of the protein

Matrix assisted laser desorption and ionization-time of flight (MALDI-TOF) mass spectrometry was employed for antimycobacterial protein identification using Bruker Daltonics Ultraflex TOF/TOF analyzer. The protein band visualized on the SDS gel was excised and the sample was subjected to in-gel digestion for analysis [42]. The sample (1µL) was mixed with α-cyano-4-hydroxycinnamic acid matrix dissolved in 70% trifluoroacetic acid (TFA) and 30% sterile purified water. The instrument was configured in positive ion mode and the spectrum was analyzed with m/z values plotted against ion intensity [35].

### In vivo toxicity studies

#### Animals

Albino Wistar rats (*Rattus norvegicus*) of either sex were chosen for acute and sub-acute toxicity studies. All the animals chosen for the study were between the ages 8 and 12 weeks with weights ranging from 120 to 250 g. The animals were obtained upon Institutional Animal Ethical Committee (IAEC) clearance (VIT/IAEC-26/17April/24/14). All the rats were housed in polycarbonate cages under standard environmental conditions in the animal facility and quarantined for a week prior to the study. All the experiments and handling of animals were carried out in accordance with the guidelines of the Organization of Economic and Co-operation and Development 423 (OECD) [43].

### Acute oral toxicity study

Acute oral toxicity study was performed to assess the single-dose toxicity of the compound. As per OECD guideline 423, female albino Wistar rats (8–12 weeks old) were chosen and the animals were divided into 4 groups with six rats per group (*n* = 6). The animals were fasted (food on hold) overnight prior to dosing. After the fasting period, the rats were weighed and administered a single dosage of 100, 350 and 700 mg/kg body weight using an oral-gavage. The control group was fed with normal saline. The animals were then fasted for 3–4 h (food on hold). They were observed for any behavioral and morphological changes for the first 30 min and thereafter for a period of 14 days. Body weight was also noted for 14 days [43].

### Sub-acute oral toxicity study

Sub-acute oral toxicity study was carried out based on the acute studies. The study was carried out adhering to the protocols briefed by OECD guidelines 407 [44]. In this study, male and female albino Wistar rats were chosen and the animals were divided into 3 groups with ten rats per group (*n* = 10; 5 males and 5 females per group). Prior to dosing, the animals were fasted (food on hold) overnight. Following the fasting period, the rats were weighed and administered doses of 100 and 350 mg/kg body weight. Doses were determined based on the absence of mortality/toxicity observed during the acute study. The control group was administered normal saline. The animals were fasted for 3–4 h (food on hold). All the animals were observed for the first 30 min for any signs of toxicity and thereafter observed daily for 28 days.

### Hematological and biochemical parameters

At the end of both studies, the animals were euthanized and blood samples were collected via cardiac puncture for assessing the toxic effects of the compound. The blood (2 mL) was collected in sterile ethylene diamine tetra acetic acid (EDTA) tubes and analyzed for hematological parameters like total red blood cell (RBC) count, total white blood cell (WBC) count, hemoglobin, packed cell volume (PCV), MCV, MCH and platelet count. For serum analysis, the blood (2 mL) was collected in serum separator tubes containing a clot activator (SSTs) and analyzed for biochemical parameters like cholesterol, high-density lipoprotein (HDL), low-density lipoprotein (LDL), total bilirubin, albumin, globulin, total protein, alkaline phosphatase (ALP), alanine aminotransferase (ALT) and aspartate aminotransferase (AST). The experiment was carried out in triplicate.

### Histopathology

After euthanizing the animals, organs such as liver, kidney, lungs and spleen were carefully extracted and immediately washed with sterile phosphate buffered saline (PBS, pH 7.4). Each of the organs was then preserved in 10% buffered formalin bottles and processed for histopathological examination. Tissue sections of 5 µm thick were embedded in paraffin wax and stained using hematoxylin–eosin stain. The sections were then mounted on resin and observed under a light microscope [45].

### Statistical analysis

All the assays were carried out in triplicates. The results were statistically analyzed using two-way ANOVA and the values were shown as mean (± SD) standard deviation which indicates the experiments were statistically significant.

## Results

### In vitro antimycobacterial activity of *Streptomyces qinglanensis* VITABS23

**Fig.1:**
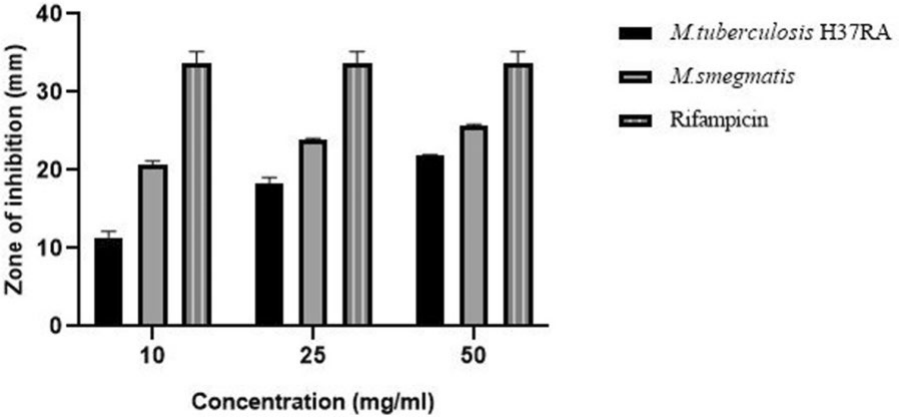
Antimycobacterial activity of *Streptomyces qinglanensis* VITABS23 in agar well diffusion assay

The cell-free extract of *Streptomyces qinglanensis* VITABS23 was screened for in vitro antimycobacterial activity using the agar well diffusion method. Among the tested concentrations (10, 25 and 50 mg/mL), the extract exhibited the maximum zone of inhibition against *M. smegmatis* (26 mm) and *M. tuberculosis* H37Ra (22 mm) at 50 mg/mL. At 25 mg/mL, moderate activity was observed against both strains, with inhibition zones of 24 mm and 19 mm, respectively (Table 1). The extract showed lower activity at 10 mg/mL, producing inhibition zones of 21 mm for *M. smegmatis* and 12 mm for *M. tuberculosis* H37Ra (Fig. 1.) To date, only a few studies have investigated marine actinobacteria for their antimycobacterial potential against *Mycobacterium* species. Therefore, marine actinobacterial extracts derived from mangrove sediments represent a valuable source of bioactive compounds with potent antimycobacterial properties.

**Table 1:** Zone of inhibition of *Streptomyces qinglanensis* VITABS23 against *Mycobacterium* strains

Isolate	Concentration (mg/mL)	Zone of inhibition (mm)	
		*M. smegmatis*	*M. tuberculosis* H37Ra
ABS23	10	21 ± 0.5	12 ± 0.7
	25	24 ± 0.2	19 ± 0.7
	50	26 ± 0.2	22 ± 0.08

Values are presented as mean ± standard deviation

### Determination of minimum inhibitory concentration (MIC)

**Fig.2:**
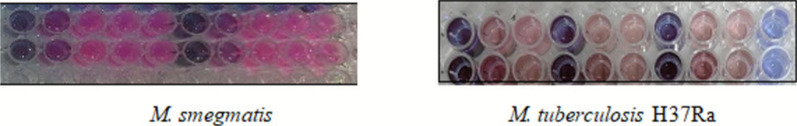
Minimum inhibitory concentration of the extract determined using MABA assay (visual determination) **a** Inhibition of *M. smegmatis* at 500 µg/ml with no color change from blue to pink. **b** Inhibition of *M. tuberculosis* H37Ra at 500 µg/ml concentration. At 200 and 125 µg/ml concentrations, a color change from blue to pink was observed for both the strains

The minimum inhibitory concentration (MIC) of the extract was determined using the Microplate Alamar Blue Assay (MABA). In this study, the inhibitory effect of the marine actinobacterial extract was evaluated against *M. smegmatis* and *M. tuberculosis* H37Ra strains. The assay was performed using three concentrations—500, 250 and 150 µg/mL each tested in triplicate. The results revealed that the *S. qinglanensis* extract exhibited significant inhibitory activity against both strains at 500 µg/mL, as evidenced by the absence of color change from blue to pink (Fig. 2.). The highest inhibitory activity was observed against *M. tuberculosis* H37Ra with 85% inhibition, while *M. smegmatis* showed 78% inhibition (Table 2).

**Table 2:** Minimum inhibitory concentration (MIC) of the marine actinobacterial extract against *M. smegmatis* and *M. tuberculosis* H37Ra strains

Sample	Concentration (µg/mL)	*M. smegmatis* (% of inhibition)	*M. tuberculosis* H37Ra (% of inhibition)
*Streptomyces qinglanensis* VITABS23	500	78%	85%

### Extraction and purification of the antimycobacterial compound

**Fig. 3:**
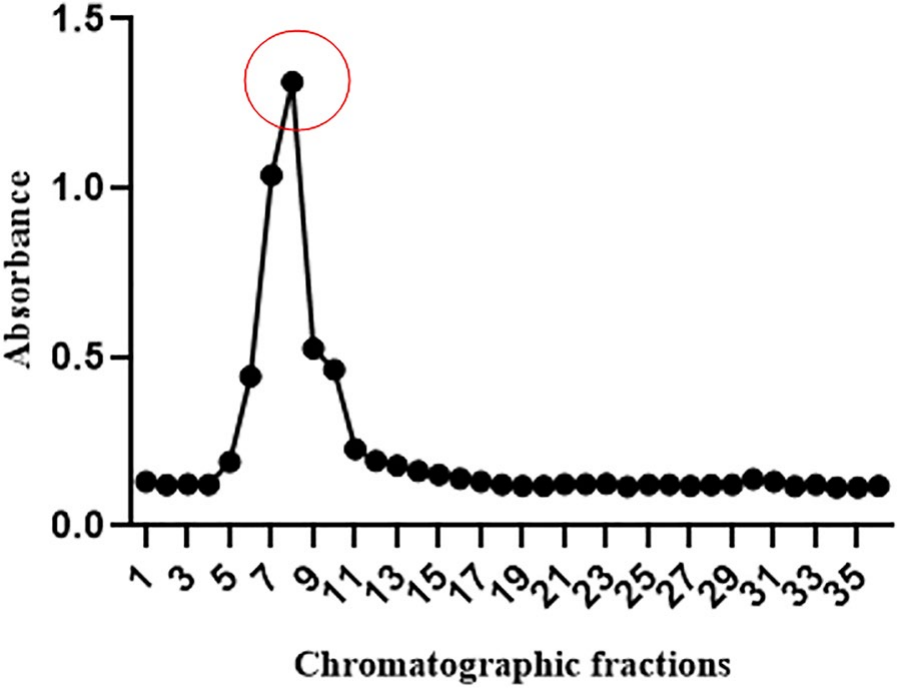
DEAE Sepharose ion exchange chromatogram showing the elution profile using 200 mM NaCl. The active fractions are marked in circle

Since *Streptomyces qinglanensis* VITABS23 exhibited promising antimycobacterial activity, it was further subjected to extraction and purification to obtain the bioactive compound. The protein of interest was precipitated using high salt gradients, thereby facilitating its separation from other biomolecules [35]. The aqueous extract obtained from solvent-solvent extraction was saturated with varying concentrations of ammonium sulphate to determine the percentage saturation of the target protein. At 40–60% ammonium sulphate saturation, the protein fractions obtained showed lower activity. When the saturation was increased to 70%, the protein fraction exhibited the highest activity (74.5%). However, the protein precipitation was observed to be the highest in 50–60% saturation; the activity was only 49.15% (Additional file 1). These results indicate that the purification process was based on bioactivity rather than yield. Further, the 70% fraction was dialyzed and subjected to ion exchange chromatography on a DEAE Sepharose Fast Flow column. The active fractions were eluted stepwise from the column using various gradients of NaCl. It was observed that the target antimycobacterial protein was eluted in the buffer containing 200 mM NaCl as shown in Fig. 3.

### Estimation of protein content and yield

The protein fractions obtained at each step were analyzed for their protein content. Purification parameters such as total protein, specific activity, percentage yield and purification fold of the antimycobacterial compound were calculated as seen in Table 3. The total inhibitory activity of the protein fraction obtained from ion exchange chromatography was 1500 IU/mL with a specific activity of 4166 IU/mg and total protein of 0.68 mg. It was noted that an increase in specific activity was followed by a decrease in total protein content resulting in a purification fold of 156, thereby indicating the purification of the antimycobacterial protein. Several antimycobacterial metabolites from marine actinobacteria have been extracted and identified through various chromatographic techniques such as GCMS and HPTLC; however no studies have reported the purification of antimycobacterial proteins using ion exchange chromatography.

**Table 3:** Purification profile of the antimycobacterial protein

Sample	Volume (mL)	Inhibitory activity (%)	Total protein (mg)	Specific activity (IU/mg)	Yield (%)	Purification fold
Crude extract	100	7600	285.3	26.63	100	1
Ammonium sulphate precipitation (60–70%)	60	4250	48.85	87.00	12.18	3.26
Dialysis	40	2800	18.54	151.02	3.41	5.67
Ion exchange fraction	30	1500	0.682	4166	0.085	156

### Characterization of the antimycobacterial compound

**Fig.4:**
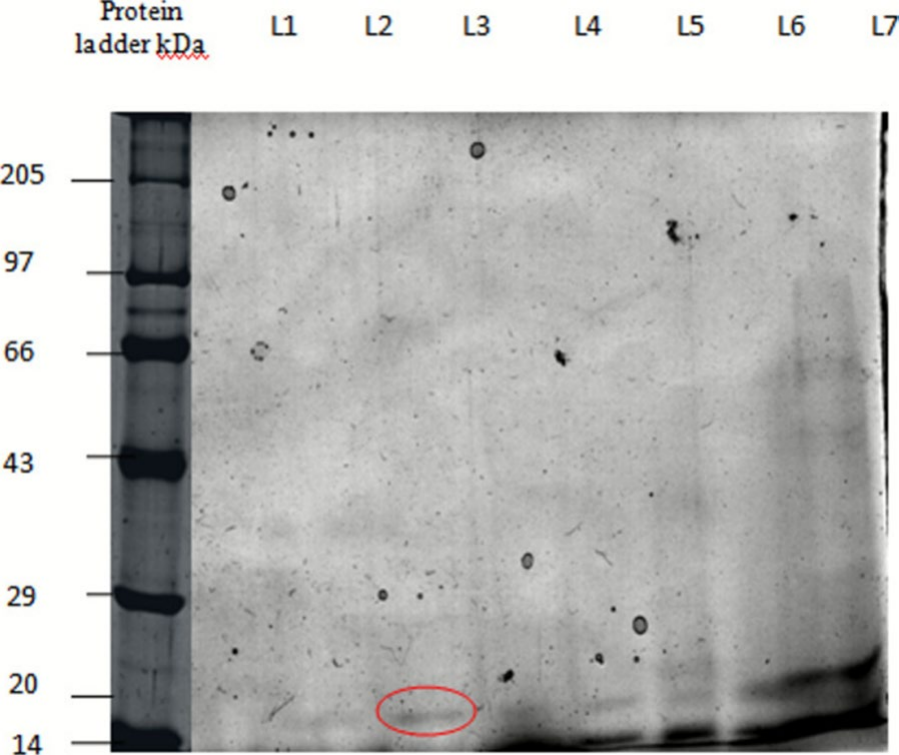
SDS PAGE profile of the antimycobacterial protein showing the presence of a single band by 200 mM elution

The molecular weight of the purified antimycobacterial compound was determined by SDS PAGE, a standard technique used in protein purification studies. In the SDS PAGE profile, purified antimycobacterial protein was analyzed together with protein ladder, crude extract, dialyzed sample and ion exchange fractions (200 mM), respectively. The broad range protein ladder comprised different molecular weight recombinant proteins ranging from 200 to 10 kDa. A single band at 20 kDa on the SDS-PAGE gel indicated the molecular weight of the purified antimycobacterial protein (Fig. 4).

### MALDI-TOF MS analysis of the protein

**Fig.5:**
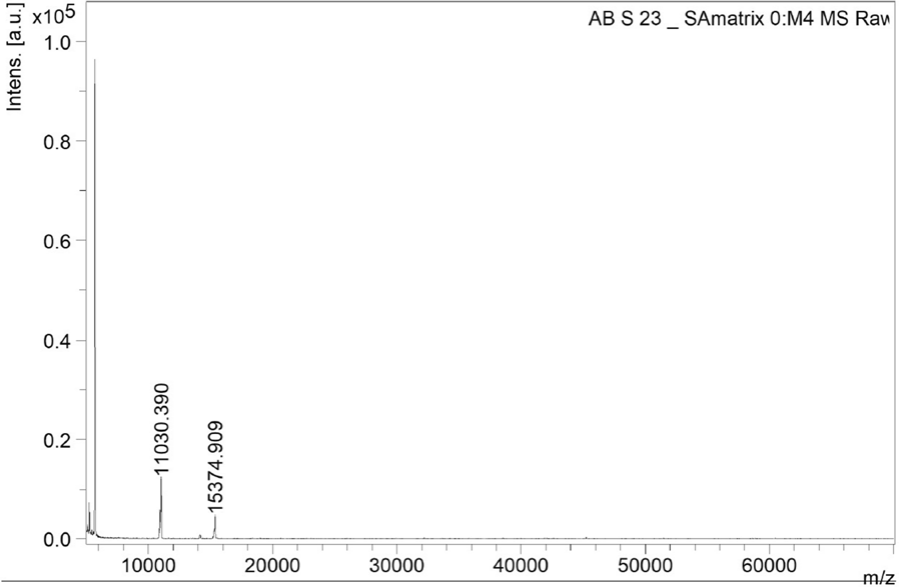
MALDI TOF MS spectrum of the antimycobacterial protein

The molecular mass profile of the purified protein fraction was analyzed using MALDI-TOF mass spectrometry. Each peak in the spectrum corresponds to a protein or peptide component present in the purified fraction (Fig. 5). The MALDI-TOF spectrum revealed two distinct peaks at approximately m/z 11,030.39 and 15,374.91 (≈ 11 kDa and 16 kDa), suggesting that the antimycobacterial compound is likely a small protein or peptide. The limited number of peaks in the spectrum indicates effective purification of the protein fraction with minimal impurities. The absence of higher m/z values beyond 20,000 suggests that the isolated protein components are predominantly of small molecular weight, a characteristic feature commonly associated with antimicrobial peptides. Further confirmation through peptide sequencing or LC–MS/MS analysis is necessary to precisely determine the protein identity.

### In vivo toxicity studies

#### Acute oral toxicity study

**Fig.6:**
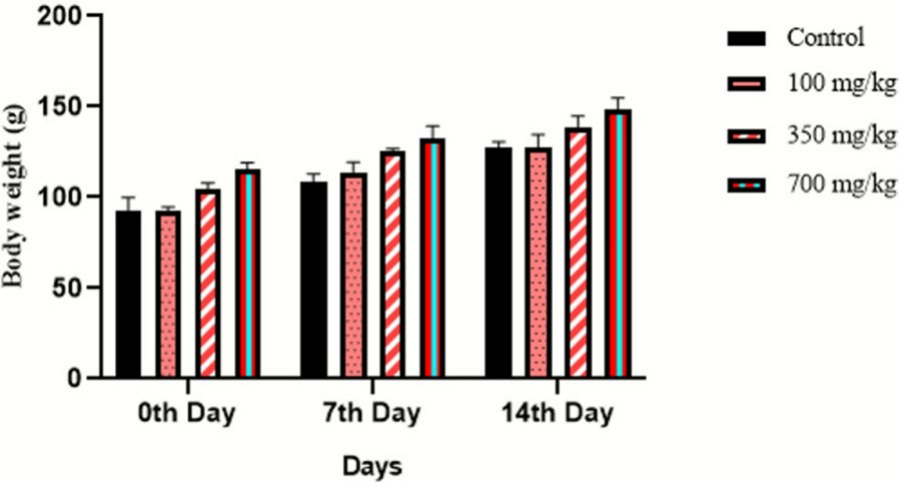
Body weight of animals during acute study following dose administration

**Fig.7:**
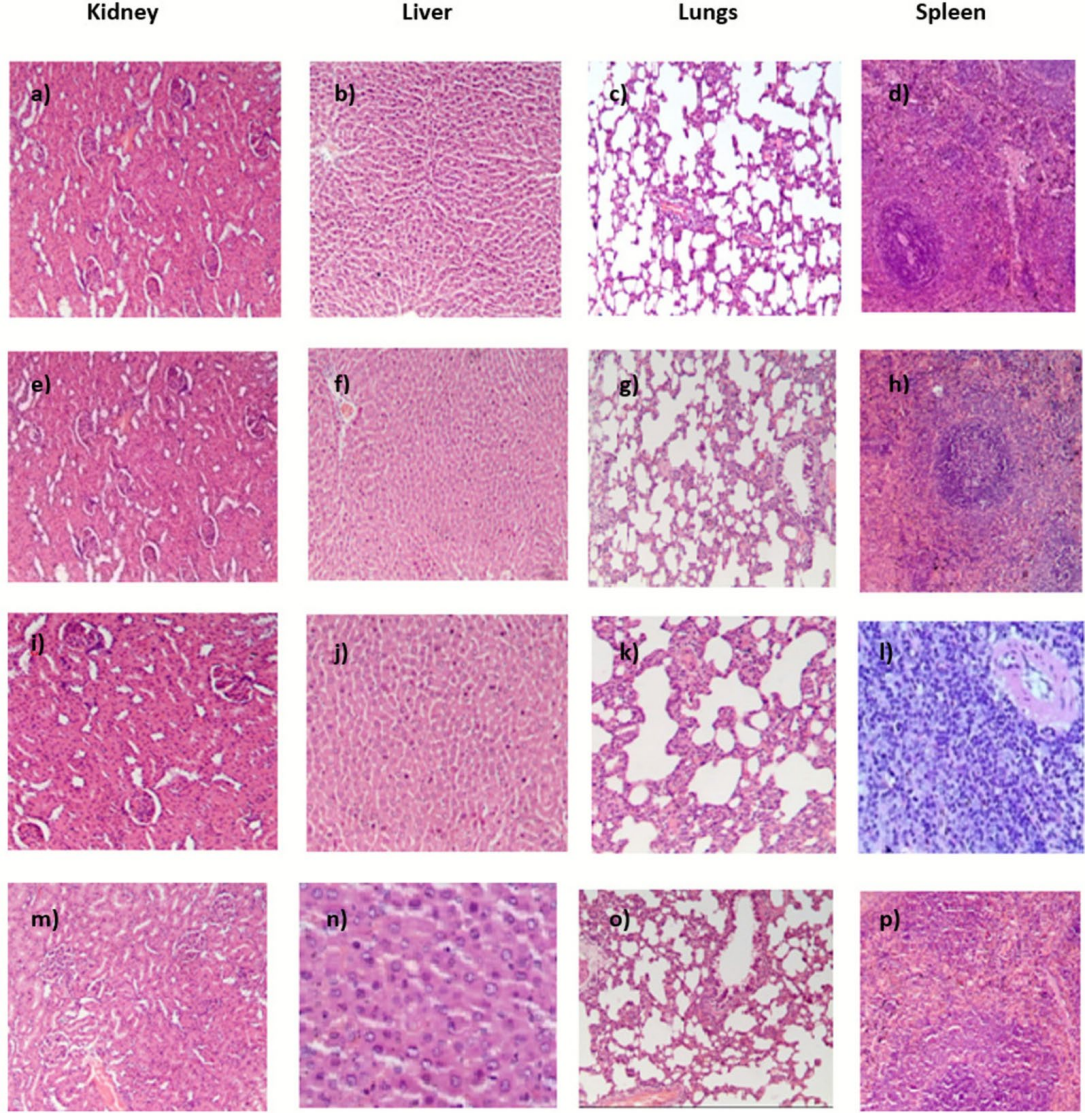
Histopathological analysis of acute toxicity study. **a** Kidney, **b** liver, **c** lungs, **d** spleen of control groups, **e** kidney, **f** liver, **g** lungs, **h** spleen of rats treated with 100 mg/kg body weight. **i** Kidney, **j** liver, **k** lungs, **l** spleen of rats treated with 350 mg/kg body weight. **m** Kidney, **n** liver, **o** lungs, p spleen of rats treated with 700 mg/kg body weight

Acute oral toxicity study was performed to assess the single-dose toxicity of the extract. This study is crucial for determining the safe dosage of the test substance in order to assess the clinical signs and symptoms associated with the drug [46]. It was observed that the marine actinobacterial extract did not show any toxic symptoms or mortalities in all the tested groups. Slight behavioral changes were noticed during the first 20 min of drug administration where the rats settled down later. It was observed that the extract did not cause any major changes in the body weight of the animals as observed in Fig. 6**.** The behavioral patterns of all the treated animals were also monitored; none of them showed any changes (Additional file 2). Hematological and serum parameters in group 1 and group 2 treated rats showed no significant variations compared to the control group. However, some adverse effects were noticed when the extract was administered in a high dosage of 700 mg/kg body weight (group 3). This can be seen from the serum and blood parameters where the cholesterol, LDL and VLDL levels were found to be 208.1 ± 4.82, 131.01 ± 0.57 and 29.28 ± 0.77 mg/dL when the extract was administered at a dosage of 700 mg/kg (Table 4). Additionally, elevated levels of liver enzymes ALT and ALP indicated possible liver damage. The WBC, PCV and MCV levels were observed to be 16.7 ± 0.45, 52.1 ± 2.94 and 73.48 ± 1.56, respectively (Table 5). These levels were quite high when compared to their reference values (Additional file 5). In toxicity studies, organs such as the liver, kidney, spleen and lungs are often affected by toxic substances [46]. Microscopic analysis of the following organs: kidney, lungs, liver and spleen of animals from the control and treated groups are shown in Fig. 7. It is evident that there were no visible hemorrhagic lesions in the control and treated rats of group 1 and group 2. Hence, these doses may not be acutely toxic to the vital organs. However, in rats treated with the 700 mg/kg dose, multifocal moderate tubular epithelial degeneration in the kidney and mild multifocal congestion in the liver were observed in rats treated with 700 mg/kg dose. Hence, the findings of the study suggest that the LD_50_ of the extract is greater than 350 mg/kg.

**Table 4:** Effect of the actinobacterial extract on serum parameters in acute toxicity study

Female rats	Doses (mg/kg body weight)			
Parameters	Control	100	350	700
Cholesterol(mg/dL)	113.04 ± 1.00	116.81 ± 1.42	126.44 ± 2.85	208.1 ± 4.82
LDL (mg/dL)	62 ± 0.19	68.21 ± 0.15	76.54 ± 0.87	131.01 ± 0.57
HDL (mg/dL)	46.71 ± 1.07	52.29 ± 0.25	57.92 ± 1.52	30.21 ± 1.02
VLDL (mg/dL)	12.78 ± 0.7	14.32 ± 0.41	16.10 ± 0.19	29.28 ± 0.77
ALP (U/L)	115.10 ± 0.95	124.51 ± 0.40	130.20 ± 2.6	150.51 ± 1.06
ALT (U/L)	43.95 ± 0.08	48.06 ± 0.16	53.25 ± 0.95	85.54 ± 0.85
AST (U/L)	143.86 ± 1.93	146.07 ± 0.88	151.41 ± 2.3	139.82 ± 1.24
Total bilirubin (mg/dL)	0.34 ± 0.08	0.40 ± 0.05	1.01 ± 0.03	1.12 ± 0.02
Globulin (g/dL)	2.8 ± 0.24	2.50 ± 0.09	2.62 ± 0.07	2.97 ± 0.06
Albumin (g/dL)	3.78 ± 0.07	4.2 ± 0.1	3.81 ± 0.07	4.6 ± 0.1
Total protein (g/dL)	7.8 ± 0.1	7.2 ± 0.1	7.63 ± 0.15	7.50 ± 0.01

*LDL* low-density lipoprotein, *HD*L high-density lipoprotein, *VLDL* very low density lipoprotein, *ALP* alkaline phosphatase, *ALT* alanine aminotransferase, *AST* aspartate aminotransferase

Results were statistically analyzed using two-way ANOVA and the values are shown as mean (± SD) standard deviation

**Table 5:** Effect of the actinobacterial extract on hematological parameters in acute study

Female rats	Doses (mg/kg body weight)			
Parameters	Control	100	350	700
Hemoglobin (g/dL)	15.16 ± 0.25	14.03 ± 0.25	14.96 ± 1.23	14.7 ± 0.55
Total WBC (10^9^/L)	09.53 ± 0.37	12.20 ± 0.51	10.83 ± 0.70	16.7 ± 0.45
Lymphocytes (%)	71.00 ± 1.006	80.53 ± 1.73	82.9 ± 2.315	75.11 ± 4.40
Monocytes (%)	3.4 ± 0.1	3.73 ± 1.10	2.8 ± 0.5	3.1 ± 043
Total RBC (10^12^/L)	8.05 ± 0.03	7.81 ± 0.36	7.80 ± 0.13	7.33 ± 0.12
P.C.V (%)	43.4 ± 0.43	43.5 ± 3.01	44.09 ± 0.37	52.1 ± 2.94
MCV (fL)	55.98 ± 0.07	54.96 ± 1.187	54.3 ± 1.59	73.48 ± 1.56
MCH (pg)	18.34 ± 0.57	18.83 ± 0.70	18.4 ± 1.04	22.66 ± 2.49
Platelet count (10^9^/L)	875.3 ± 4.04	813 ± 10	754 ± 6	767 ± 7
MPV (fL)	5.56 ± 0.15	5.73 ± 03	5.76 ± 0.40	5.2 ± 0.15

*WBC* white blood cells, *RBC* red blood cells, *P.C.V* packed cell volume, *MCV* mean corpuscular volume, *MCH* mean corpuscular hemoglobin, *MP*V mean platelet volume

Results were statistically analyzed using two-way ANOVA and the values are shown as mean (± SD) standard deviation

#### Sub-acute oral toxicity study

**Fig.8:**
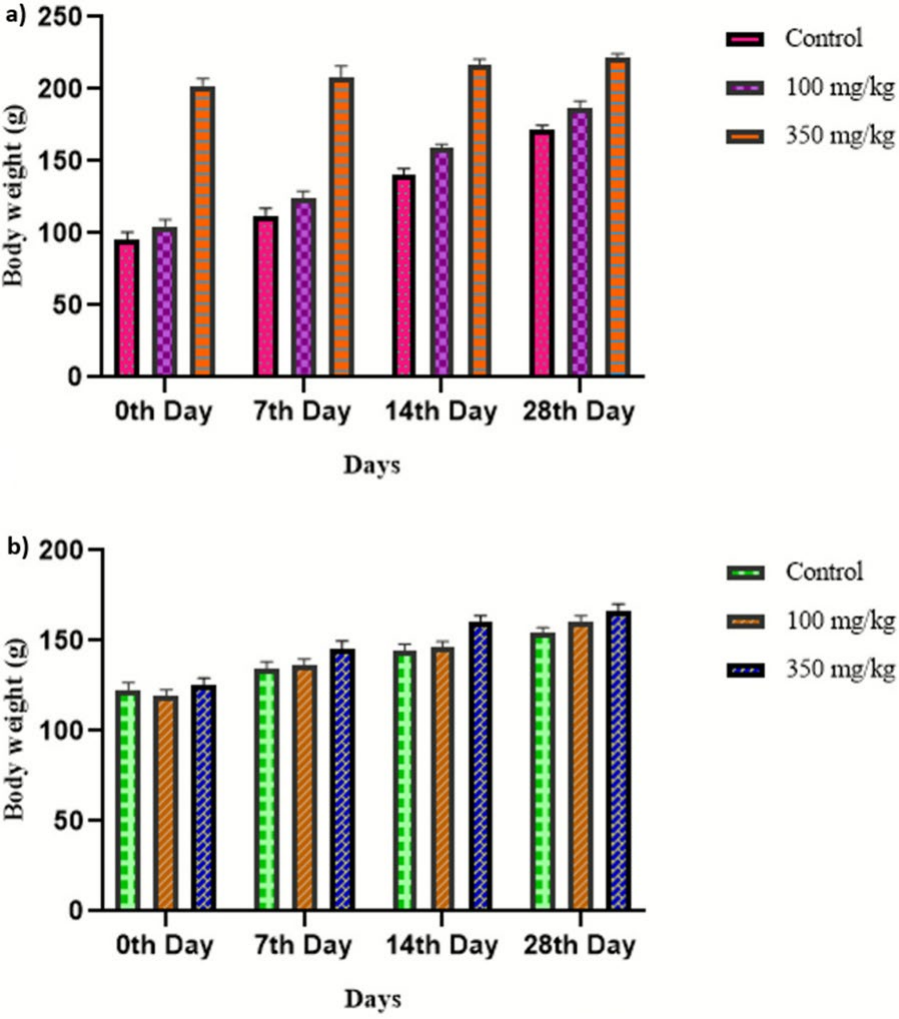
Body weight of animals during sub-acute study following dose administration: **a** males, **b** females

**Fig. 9:**
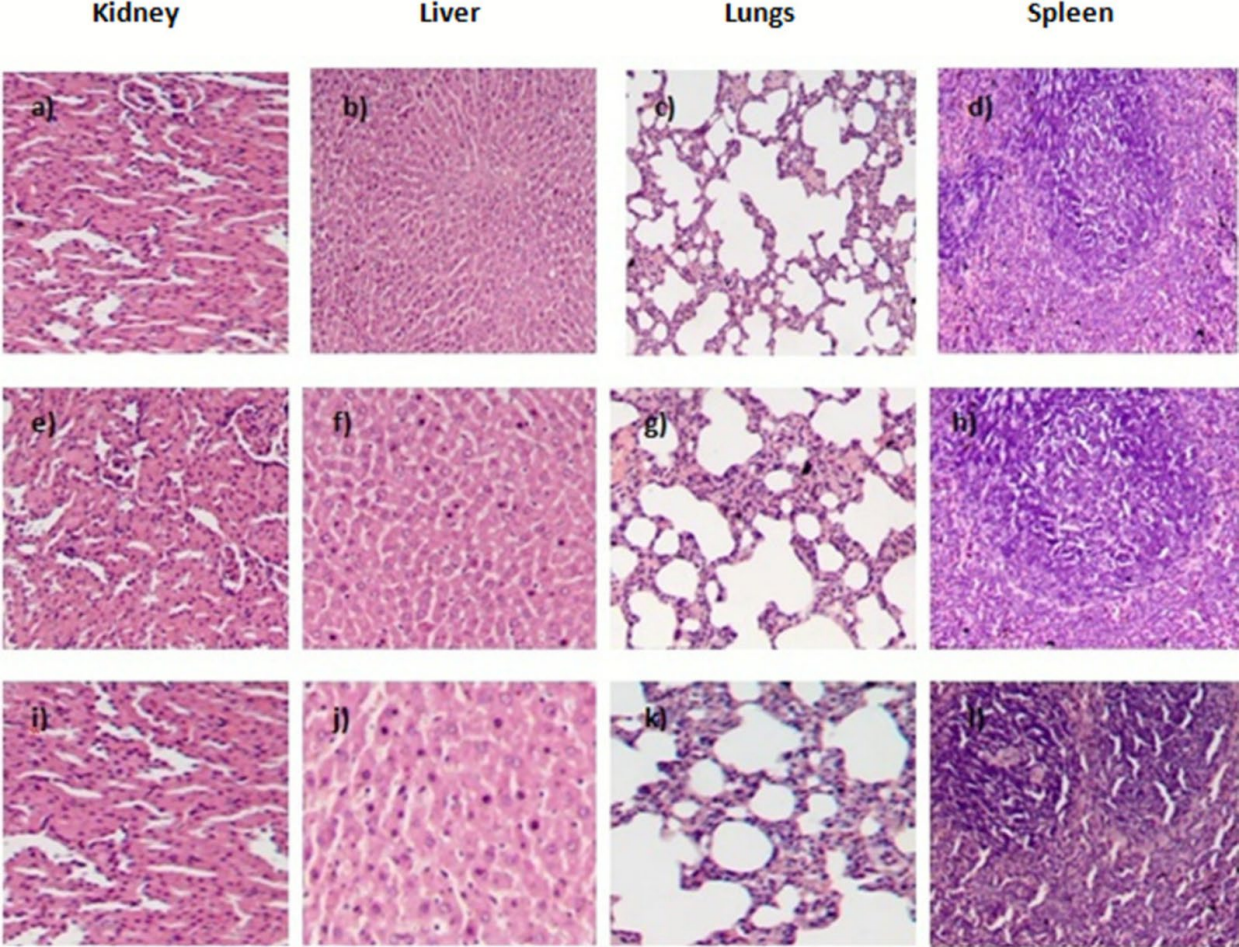
Histopathological analysis of sub-acute toxicity study in males. **a** Kidney, **b** liver, **c** lungs, **d** spleen of control groups. **e** Kidney, **f** liver, **g** lungs, **h** spleen of rats treated with 100 mg/kg body weight. **i** Kidney, **j** liver, **k** lungs, **l** spleen of rats treated with 350 mg/kg body weight

As no toxic effects were observed in the acute study at 100 and 350 mg/kg body weight, a sub-acute study was carried out in both males and females to further evaluate toxicity. In vivo toxicity assays are widely used to determine the safe therapeutic doses for drug formulations [47]. In this study, no significant changes were observed in the overall morphological and behavioral patterns of treated groups when compared to the control, following daily oral administration of the extract at doses of 100 and 350 mg/kg body weight for a period of 28 days. Throughout the treatment period, both control and treated rats seemed healthy and no signs of mortality were observed. No alterations were observed in food intake and sleep pattern (Additional file 3). The weekly body weights of both male and female rats increased throughout the experiment, with the males heavier than the females (Fig. 8). Also, sub-acute oral administration of the extract did not result in variation in the hematological and serum parameters in both control and treated groups (Table 6 and Table 7). Comparing the hematological parameters of both males and females shows that lymphocytes, RBC, and PCV were slightly higher in males whereas WBC and MPV were higher in females (Additional file 6). However, their mean values remained within normal ranges compared with the control groups (Table 8 and Table 9). The organs of the treated rats showed no visible changes in color or texture compared to the control group. Microscopic analysis of the organs (Kidney, Liver, Lungs and Spleen) showed no significant lesions or necrosis in either control or treated groups (both male and female) (Fig. 9) (Additional file 4). These results suggest that the marine actinobacterial extract is unlikely to contain toxic compounds, indicating its safety.

**Table 6:** Effect of the actinobacterial extract on serum parameters of sub-acute toxicity study in males

Male rats	Doses (mg/kg body weight)		
Parameters	Control	100	350
Cholesterol (mg/dL)	127.37 ± 1.15	125.51 ± 1.46	129.5 ± 1.05
LDL (mg/dL)	48.9 ± 0.2	42.52 ± 0.58	46.25 ± 0.83
HDL (mg/dL)	39.6 ± 0.52	37.16 ± 1.46	37.36 ± 1.03
VLDL (mg/dL)	16.99 ± 0.01	16.06 ± 0.71	16.76 ± 0.21
ALP (U/L)	140.7 ± 0.60	137.15 ± 2.03	139.43 ± 0.56
ALT (U/L)	38.01 ± 0.18	34.22 ± 2.06	37.01 ± 1.48
AST (U/L)	149.96 ± 0.05	142.37 ± 2.54	147.43 ± 1.02
Total bilirubin (mg/dL)	1.19 ± 0.01	1.08 ± 0.02	1.09 ± 0.01
Globulin (g/dL)	2.7 ± 0.1	2.16 ± 0.20	2.36 ± 0.15
Albumin (g/dL)	4.96 ± 0.11	4.38 ± 0.17	5 ± 0.1
Total protein (g/dL)	7.6 ± 0.1	7.7 ± 0.1	7.6 ± 0.1

Results were statistically analyzed using two-way ANOVA and the values are shown as mean (± SD) standard deviation

**Table 7:** Effect of the actinobacterial extract on serum parameters of sub-acute toxicity study in females

Female rats	Doses (mg/kg body weight)		
Parameters	Control	100	350
Cholesterol (mg/dL)	120.22 ± 0.52	119.56 ± 0.69	118.63 ± 0.90
LDL (mg/dL)	47.74 ± 0.94	44.173 ± 1.15	46.33 ± 0.56
HDL (mg/dL)	48.26 ± 0.58	37.36 ± 1.03	43.50 ± 0.91
VLDL (mg/dL)	16.73 ± 0.15	16.09 ± 0.66	16.91 ± 0.03
ALP (U/L)	138.42 ± 1.70	130.45 ± 0.65	134.39 ± 1.10
ALT (U/L)	35.57 ± 0.85	37.21 ± 0.23	38.28 ± 0.58
AST (U/L)	143.84 ± 1.02	139.53 ± 1.00	143.32 ± 1.02
Total bilirubin (mg/dL)	1.05 ± 0.01	0.99 ± 0.01	1.03 ± 0.05
Globulin (g/dL)	3.43 ± 0.30	3.23 ± 0.15	3.4 ± 0.1
Albumin (g/dL)	4.63 ± 0.15	4.66 ± 0.05	4.5 ± 0.1
Total protein (g/dL)	7.4 ± 0.2	7.4 ± 0.1	7.46 ± 0.05

Results were statistically analyzed using two-way ANOVA and the values are shown as mean (± SD) standard deviation

**Table 8:** Effect of the actinobacterial extract on hematological parameters of sub-acute toxicity study in males

Males	Doses (mg/kg body weight)		
Parameters	Control	100	350
Hemoglobin (g/dL)	14.12 ± 0.593	15.6 ± 0.2	15.01 ± 0.68
Total WBC (10^9^/L)	12.033 ± 0.96	10.8 ± 0.37	11.21 ± 0.76
Lymphocytes (%)	72.67 ± 0.31	73.04 ± 0.94	78.96 ± 2.4
Monocytes (%)	4.7 ± 0.1	4.2 ± 0.1	4.56 ± 0.32
Total RBC (10^12^/L)	8.44 ± 0.05	7.78 ± 0.46	8.33 ± 0.08
P.C.V (%)	44.27 ± 0.70	44.23 ± 0.77	46.04 ± 0.12
MCV (fL)	55.9 ± 0.65	61.18 ± 0.96	62.2 ± 0.52
MCH (pg)	18.33 ± 0.15	17.82 ± 0.26	18.96 ± 0.56
Platelet count (10^9^/L)	913.66 ± 18.21	736.9 ± 25.08	928.3 ± 58.85
MPV(fL)	6.93 ± 0.35	6.56 ± 0.35	6.66 ± 0.23

*WBC* white blood cells, *RBC* red blood cells, *P.C.V* packed cell volume, *MCV* mean corpuscular volume, *MCH* mean corpuscular hemoglobin, *MP*V mean platelet volume

Results were statistically analyzed using two-way ANOVA and the values are shown as mean (± SD) standard deviation

**Table 9:** Effect of the actinobacterial extract on hematological parameters of sub-acute toxicity study in females

Females	Doses (mg/kg)		
Parameters	Control	100	350
Hemoglobin (g/dL)	14.05 ± 0.04	13.84 ± 0.24	14.07 ± 0.05
Total WBC (10^9^/L)	12.04 ± 0.02	11.38 ± 0.12	11.46 ± 0.15
Lymphocytes (%)	70.65 ± 0.45	65.16 ± 0.99	67.36 ± 1.44
Monocytes (%)	4.88 ± 0.02	3.8 ± 0.1	4.10 ± 0.08
Total RBC (10^12^/L)	7.89 ± 0.02	7.52 ± 0.11	7.78 ± 0.14
P.C.V (%)	44.57 ± 0.50	44.1 ± 0.4	55.86 ± 0.05
MCV (fL)	56.97 ± 0.55	54.52 ± 0.97	55.86 ± 0.05
MCH (pg)	18.35 ± 0.55	17.97 ± 0.06	18 ± 0.1
Platelet count (10^9^/L)	441.03 ± 0.95	438.00 ± 12.43	475 ± 13.22
MPV (fL)	7.22 ± 0.38	6.78 ± 0.29	7.86 ± 0.35

Results were statistically analyzed using two-way ANOVA and the values are shown as mean (± SD) standard deviation

## Discussion

The study focuses on the antimycobacterial and toxicological properties of the aqueous extract derived from *Streptomyces qinglanensis* VITABS23, isolated from mangrove sediments of Kerala. Mangroves are specialized coastal ecosystems that support a rich diversity of flora and fauna [48]. The microbial communities thriving in this habitat are well known for producing unique secondary metabolites with multifunctional activities such as antibacterial, antioxidant, antiviral, anti-inflammatory, and antifungal properties [49]. Among them, actinobacteria are recognized as prolific producers of bioactive compounds with significant therapeutic potential. However, the antimycobacterial activity and safety assessment of mangrove-derived actinobacteria remain poorly explored. To our knowledge, this is the first report describing the antimycobacterial and toxicological evaluation of *S. qinglanensis* VITABS23 against *Mycobacterium tuberculosis*. The results of the study showed that the cell-free extract of *S. qinglanensis* demonstrated notable in vitro antimycobacterial activity, showing inhibition zones of 26 mm and 22 mm against *M. smegmatis* and *M. tuberculosis* H37Ra, respectively, at a 50 mg/mL concentration. These inhibition zones were comparatively higher than those reported for other actinobacterial strains. Huynh et al. (2024) evaluated the antimycobacterial potential of *Streptomyces alboniger* A121 against *M. smegmatis* and reported a maximum inhibition zone of 15 mm [22]. Similarly, Kurnijasanti et al. (2023) investigated soil-derived *Streptomyces* isolates from Indonesia against *M. tuberculosis* H37Rv and observed inhibition zones of 15 ± 0.9 mm, 8 ± 0.7 mm and 2 ± 0.3 mm, respectively [30]. In another study, Manigundan et al. (2019) investigated the antimycobacterial potential of *Streptomyces* sp. isolated from the Andaman mangrove sediments against *M.* smegmatis. The extract exhibited a maximum inhibition zone of 13 mm, indicating moderate activity [50]. In comparison, *Streptomyces qinglanensis* VITABS23 exhibited substantially greater inhibitory activity, producing zones of inhibition of 26 mm against *M. smegmatis* and 22 mm against *M. tuberculosis* H37Ra. These findings suggest that *S. qinglanensis* VITABS23 synthesizes potent antimycobacterial metabolites, underscoring its potential as a promising source of novel antimycobacterial agents. Minimum inhibitory concentration (MIC) testing using the Microplate Alamar Blue Assay (MABA) further confirmed this activity. MABA is a quantitative method used to measure the drug susceptibility in bacteria. This method employs the use of Alamar Blue, which undergoes a color change from blue to pink upon oxidation–reduction reaction, indicating the tested drug is ineffective against the bacterial strains whereas the absence of color change signifies inhibition of bacteria [32, 51]. The MIC of the extract was determined to be 500 µg/mL, where the highest inhibitory activity was observed against *M. tuberculosis* H37Ra, with 85% inhibition, while *M. smegmatis* exhibited 78% inhibition. In a study reported by ArokiaRajan et al. (2023), the inhibitory potential of a marine seaweed extract against *M. smegmatis* was assessed using the microplate Alamar Blue Assay at 250 and 500 µg/mL concentrations, where significant bacterial growth inhibition was observed [52]. Similarly, Hussain et al. (2022) evaluated the antimycobacterial activity of the rare actinobacteria *Lentzea violacea* against *M. tuberculosis* H37Rv and *M. smegmatis.* The*.* crude extracts of *L. violacea* exhibited strong inhibitory activity against *M. tuberculosis* H37Rv, with MIC values ranging from 62.5 to 250 µg/mL [42]. In another study, Nekkanti et al. (2021) reported that the ethyl acetate extract of *Streptomyces* sp. MCA2 showed 52.49% inhibition against *M. tuberculosis* H37Rv at 500 µg/mL, while only 40% inhibition was observed at 100 µg/mL [53]. These findings of the present study are therefore in accordance with these previous reports, further supporting the antimycobacterial potential of marine-derived actinobacterial extracts.

In this context, *Streptomyces qinglanensis* VITABS23 was further subjected to extraction and purification to obtain the bioactive compound. The antimycobacterial compound was purified using ion exchange chromatography, achieving a 156-fold purification, with a protein yield of 0.085% and a specific activity of 4166 IU/mg. In comparison, Ismail et al. (2024), reported that the purification of an antimycobacterial compound using DEAE cellulose column chromatography resulted in a protein yield of 43.9% with 28.4% total protein recovery [54], which is comparatively higher than that observed in the present study. However, Kamarudheen et al. (2021) achieved a 230-fold purification with a protein yield of 0.097% for an antiviral protease inhibitor, which is comparable to our finding [35]. Similarly, Sarkar et al. (2023) purified a serine protease inhibitor with a purification fold of 65.7 and a yield of 38% [33]. These findings indicate that both protein yield and purification fold are influenced by the specific characteristics of the compound being purified. The relatively lower yield observed in this study could be attributed to its partial loss during multiple purification steps, whereas the 156-fold increase in specific activity indicates that the target protein has been successfully enriched and concentrated. SDS-PAGE analysis of the purified protein revealed an approximate molecular weight of 20 kDa, while MALDI-TOF analysis indicated a molecular mass in the range of 11–16 kDa, which closely correlates with the SDS-PAGE result. This suggests that the purified compound is a small protein or peptide with potential antimycobacterial activity. However, the present study has certain limitations. Although the purified compound was characterized by SDS-PAGE and MALDI-TOF analyses, its complete amino acid sequence and structural identity were not determined. Advanced proteomic analyses such as LC–MS/MS would be necessary to confirm the exact protein sequence and elucidate its functional domains. Furthermore, the mechanism underlying its antimycobacterial activity remains to be elucidated. Future studies should focus on identifying the molecular targets, evaluating the stability and bioavailability of the compound under physiological conditions, and assessing its synergistic potential with existing antimycobacterial drugs. Based on previous studies, antimycobacterial compounds derived from marine actinobacteria are known to act through multiple mechanisms. Compounds such as thiolactomycin and pyridomycin from *Streptomyces* sp. target the cell wall of *M. tuberculosis* by inhibiting fatty acid synthase II (FAS II) enzymes including InhA, KasA and KasB. In contrast, other compounds such as rifamycin, kanamycin and rifabutin interfere with the intracellular processes, including nucleic acid and protein synthesis [55]. Additionally, cyclomarins, peptide metabolites derived from marine actinobacteria, bind to the catalytic center of the ClpC1 component of the protease complex, thereby disrupting protein homeostasis [55, 56]. Considering these diverse modes of action, it is assumed that the present protein may also function through any one or more of these mechanisms. Further biochemical validation and molecular docking studies will be essential to characterize its precise molecular targets and define its mode of action against *M. tuberculosis*.

Toxicological evaluation is a crucial step in determining the safety of microbial secondary metabolites, as certain metabolites are known to exhibit toxic effects [57]. In the drug discovery process, understanding the pharmacodynamic and pharmacokinetic properties of bioactive compounds is essential, but equal emphasis must be placed on evaluating their safety profiles [58]. Unfortunately, limited information is available on the toxicity profiles of secondary metabolites derived from microbial sources. Therefore, the present study was conducted to assess the toxicity profile of the marine actinobacterial extract in animal models, following the guidelines of the Organization of Economic and Co-operation and Development (OECD) 423. Toxicity studies not only provide insights into the adverse effects of test substances but also help determine their safe dose ranges [46, 59]. In the present study, both acute and sub-acute toxicity assessments of the marine actinobacterial extract were carried out in albino Wistar rats.

Following oral administration, the animals were observed for behavioral and morphological changes, and further evaluated through biochemical, hematological and histopathological analyses. In the acute toxicity test, single-dose administration at 100 mg/kg and 350 mg/kg of the extract did not produce any signs of toxicity or mortality in the treated groups. However, rats treated with 700 mg/kg exhibited some adverse effects as reflected in biochemical, hematological and histopathological analyses. Hence, the LD_50_ of the extract was estimated to be greater than 350 mg/kg. Similarly, the sub-acute toxicity study carried out with two doses (100 and 350 mg/kg) for a period of 28 days revealed no observable toxic symptoms. Oral administration of the extract did not result in significant alterations in hematological or serum parameters in either the control or treated groups. Histopathological analysis of the vital organs—including the kidney, liver, lungs, and spleen—showed no lesions, necrosis or other pathological changes. In toxicity studies, vital organs such as the liver and kidneys play a critical role in detoxification and eliminating chemicals [59–61]. Therefore, the evaluation of these two organs plays a critical role in assessing safety and predicting potential drug failures. These findings suggest that the marine actinobacterial extract is unlikely to contain toxic compounds, indicating its safety. In agreement with our observations, Gomathi et al. (2019) evaluated the toxicity of a mangrove actinobacterial extract in animal models and reported no adverse effects on hepatic enzyme levels at doses of 250 and 500 mg/kg body weight, further confirming its safety [62]. However, the present investigation provides only a preliminary in vivo toxicity assessment based on acute and sub-acute exposure. Chronic and long-term toxicity studies are required to fully establish the safety profile of the extract. Such studies will help validate the therapeutic potential of *S. qinglanensis* VITABS23 as a safe and effective source of novel antimycobacterial agents.

## Conclusion

Marine actinobacteria from mangrove sediments are nature’s molecular architects, producing compounds with potent antimycobacterial activity. The phylum Actinobacteria has played a distinguished role in the discovery of antimycobacterial drugs. The findings of this study demonstrate that *Streptomyces qinglanensis* VITABS23 exhibits promising antimycobacterial activity, suggesting its potential as a new lead for TB drug development. The 20 kDa purified protein showed significant antimycobacterial activity against *M. tuberculosis* H37Ra and *M. smegmatis* strains. Furthermore, the acute and sub-acute toxicity studies of the aqueous extracts revealed no toxic effects, supporting its safety for future tuberculosis treatment. However, identifying the molecular targets of the purified protein and conducting in vivo efficacy studies are necessary to validate its therapeutic effectiveness and advance its development as a potential drug candidate.

## Supplementary Information

Supplementary Material 1.

Supplementary Material 2.

Supplementary Material 3.

Supplementary Material 4.

Supplementary Material 5.

Supplementary Material 6.

## Data Availability

Data will be made available on request.

## Abbreviations

COVID-19: Coronavirus disease-19
NaCl: Sodium chloride
HCl: Hydrochloric acid
V: Voltage
h: Hours
ANOVA: Analysis of variance
MALDI-TOF: Matrix assisted laser desorption/ionization-time of flight
m/z: Mass-to-charge ratio
LD: Lethal dose
