# Supplementary material for: Investigation of the antimycobacterial potential and toxicity evaluation of a proteinaceous compound from Streptomyces qinglanensis VITABS23 against Mycobacterium tuberculosis strains
Source: Trop Med Health. 2025 Dec 19;53:191. doi: 10.1186/s41182-025-00859-6 (PMC12720479; doi:10.1186/s41182-025-00859-6)
Supplement: Supplementary file 4 — Supplementary Material 4. [file 41182_2025_859_MOESM4_ESM.docx]

**Additional file 4. Behavioral and morphological characteristics in animals over 28 days**

| **Parameters** | **30 min** | **24 hours** | **Day 7** | **Day 14** | **Day 28** |
| --- | --- | --- | --- | --- | --- |
| Hyperactivity | Slight | NO | NO | NO | NO |
| Drowsiness | NO | NO | NO | NO | NO |
| Tremors | NO | NO | NO | NO | NO |
| Breathing pattern | Normal | Normal | Normal | Normal | Normal |
| Sleep | - | Normal | Normal | Normal | Normal |
| Salivation | Normal | Normal | Normal | Normal | Normal |
| Urination | - | Normal | Normal | Normal | Normal |
| Food intake | - | Usual | Usual | Usual |  |
| Diarrhea | NO | NO | NO | NO | NO |
| Skin and fur | Piloerection | Normal | Normal | Normal | Normal |
| Eyes | Normal | Normal | Normal | Normal | Normal |
| Coma | NO | NO | NO | NO | NO |
